# Choroidal involvement in systemic vasculitis: a systematic review

**DOI:** 10.1186/s12348-022-00292-4

**Published:** 2022-04-04

**Authors:** Pınar Çakar Özdal, Ilknur Tugal-Tutkun

**Affiliations:** 1 Department of Ophthalmology, University of Health Sciences, Ulucanlar Eye Education and Research Hospital, Ankara, Turkey; 2 Department of Ophthalmology, Istanbul Faculty of Medicine, Istanbul University, Istanbul, Turkey

**Keywords:** Systemic vasculitis, Choroidal involvement, Ischemia, Inflammation, Multimodal imaging

## Abstract

Systemic vasculitides are a large group of heterogeneous diseases characterized by inflammatory destruction of blood vessels targeting diverse organs and tissues including the eye. As the most vascularized layer of the eye, the choroid is expected to be affected in multiple systemic rheumatologic diseases with vascular involvement. While there are plenty of studies investigating retinal vascular involvement, choroidal vascular involvement in systemic vasculitides has not been investigated in isolation. However, choroidal manifestations including thickness changes, choroidal vasculitis and ischemia may be the earliest diagnostic features of systemic vasculitic diseases. Thus, multimodal imaging of the choroid may help early detection of choroidal involvement which may also have prognostic implications in these life-threatening diseases. This article aimed to review involvement of the choroid in systemic vasculitic diseases.

## Introduction

Systemic vasculitides are a large group of heterogeneous diseases characterized by inflammatory disruption of blood vessels which target diverse organs and tissues including the eye [1]. As various-sized vessels throughout the body may be involved, the classification of systemic vasculitis is mainly based on vessel size [2]. The first classification made in 1994, was later revised at the International Chapel Hill Consensus Conference held in 2012 (CHCC 2012). CHCC 2012 has been described as a nomenclature system rather than being a classification or diagnostic system [3]. The vessel involvement in systemic vasculitis is not restricted to only a single vessel size but named after the predominantly involved vessel. In a large vessel vasculitis the characteristic feature is the inflammation of the large vessels; however, medium and small sized vessels may also be affected. This revised nomenclature of systemic vasculitides is shown in Table 1.

**Table 1:** Classification of systemic vasculitides according to CHCC nomenclature [3]

**Large vessel vasculitis (LVV)**	
• Takayasu arteritis (TA)	
• Giant cell arteritis (GCA)	
**Medium vessel vasculitis (MVV)**	
• Polyarteritis nodosa (PAN)	
• Kawasaki disease (KD)	
**Small vessel vasculitis (SVV)**	
• Antineutrophil cytoplasmic antibody (ANCA)–associated vasculitis (AAV)	
◦ Microscopic polyangiitis (MPA)	
◦ Granulomatosis with polyangiitis (Wegener’s) (GPA)	
◦ Eosinophilic granulomatosis with polyangiitis (Churg-Strauss) (EGPA)	
• Immune complex SVV	
◦ Anti–glomerular basement membrane (anti-GBM) disease	
◦ Cryoglobulinemic vasculitis (CV)	
◦ IgA vasculitis (Henoch-Schönlein) (IgAV)	
◦ Hypocomplementemic urticarial vasculitis (HUV) (anti-C1q vasculitis)	
**Variable vessel vasculitis (VVV)**	
• Behçet’s disease (BD)	
• Cogan’s syndrome (CS)	
**Single-organ vasculitis (SOV)**	
• Cutaneous leukocytoclastic angiitis	
• Cutaneous arteritis	
• Primary central nervous system vasculitis	
• Isolated aortitis	
• Others	
**Vasculitis associated with systemic disease**	
• Lupus vasculitis	
•Rheumatoid vasculitis	
•Sarcoid vasculitis	
• Others	
**Vasculitis associated with probable etiology**	
• Hepatitis C virus–associated cryoglobulinemic vasculitis	
• Hepatitis B virus–associated vasculitis	
• Syphilis-associated aortitis	
•Drug-associated immune complex vasculitis	
• Drug-associated ANCA-associated vasculitis	
• Cancer-associated vasculitis	
• Others	

Because the inflammation and destruction of the blood vessels may cause tissue or end organ ischemia, systemic vasculitides are a group of diseases with life-threatening consequences [1, 2]. It has been reported that patients with ocular manifestations of antineutrophil cytoplasmic antibody (ANCA)–associated vasculitis (AAV) experienced 2.75-fold higher mortality than patients with other inflammatory eye diseases. Therefore, the ability to recognize ocular manifestations of vasculitis and associating them with the underlying systemic disease is very important in order to prevent morbidity and mortality [4].

Ocular involvement may be present at the time of diagnosis, may develop later during the course, or may even be the first manifestation of a potentially life-threatening condition, needing a close collaboration between ophthalmologists and other specialists [1]. Ocular manifestations vary depending on the underlying systemic vasculitis. In a study which aimed to determine the frequencies and types of ophthalmic manifestations among 1286 patients with systemic necrotizing vasculitis, 214 (16.6%) had ocular involvement at diagnosis. Among patients with ophthalmologic manifestations, the most common types of involvement were conjunctivitis (42%), episcleritis (26%), and orbital inflammation (12%) [5]. Ocular manifestations were significantly more often in granulomatosis with polyangiitis (GPA) (34.1%) than eosinophilic granulomatosis with polyangiitis (EGPA) (11.1%), polyarteritis nodosa (PAN) (10.7%) or microscopic polyangiitis (MPA) (8.9%) [5]. Orbital inflammation is one of the most common manifestations of AAV and may present with orbital pain, eyelid edema, restricted eye movements, diplopia, epiphora and proptosis [1, 6, 7]. Patients may also present with painful and red eyes due to conjunctivitis, episcleritis, scleritis, peripheral ulcerative keratitis or anterior uveitis [1, 4, 5, 8, 9]. Another less common manifestation is a sudden visual loss which may be associated with ischemic optic neuropathy due to occlusion of posterior ciliary arteries, optic nerve compression, or retinal vascular occlusions [1, 5, 10, 11]. Since all structures of the eye and orbital tissues may be involved in systemic vasculitis, the term “ocular vasculitis” was proposed by Herbort et al. which includes episcleritis, scleritis, peripheral ulcerative keratitis, retinal vasculitis, choroidal vasculitis, optic nerve vasculitis, as well as orbital and adnexal lesions [12].

The causes and systemic associations as well as clinical signs and diagnostic ocular imaging of retinal vasculitis and occlusive retinal vasculopathy have been extensively studied in the literature [13–18]. In large series of retinal vasculitis, an association has been rarely found with large or medium vessel vasculitis or AAV [14, 15]. Rosenbaum et al. reported that systemic vasculitis accounted for only 1.4% of 207 cases of retinal vasculitis [14]. Behçet’s disease, sarcoidosis, and systemic lupus erythematosus (SLE) are more common systemic associations of retinal vasculitis [14–16]. The differential diagnosis is guided by ocular clinical findings as well as fluorescein angiography (FA) to identify retinal arterial versus venous involvement and occlusive versus nonocclusive nature of retinal vasculitis [17, 18]. Unlike the abundance of studies of retinal vasculitis in the literature, choroidal vasculitis has not been investigated in isolation. There are recent reviews on classification and imaging of immune mediated choroiditis including choriocapillaropathies [19, 20] and ischemic choroidal diseases [21]; however, there is a paucity of data on the involvement of choroidal vessels in systemic vasculitides. The aim of this article was to review involvement of the choroid in systemic vasculitic diseases.

### Choroidal circulation and ophthalmoscopic signs of choroidal vasculopathy

The choroid is a highly vascularized and pigmented tissue supplied by branches of the posterior and anterior ciliary arteries which originate from the ophthalmic artery, the first branch of the internal carotid artery. The short posterior ciliary arteries give rise to the major choroidal arterioles. Recurrent branches of the long posterior ciliary arteries as well as anterior ciliary arteries contribute to the anterior choroidal blood supply. Branches of ciliary arteries lying in Haller’s layer (posterior choroidal stroma) give rise to the medium-sized choroidal arterioles of the Sattler’s layer (anterior choroidal stroma), which in turn supply the choriocapillaris, a single layer of choroidal capillaries containing fenestrated endothelium. The circulation in the choroid is segmental [22]. The choriocapillaris contains numerous anastomotic vessels, but is functionally divided into lobules, with a central arteriolar feeder and an array of draining venules. The outer choroidal stroma containing large vessels is mainly a venous system, coalescing into 4–8 vortex veins that drain into the superior and inferior orbital veins [22]. Being the most vascularized layer of the eye supplying nutrients to the outer retina, the choroid contributes to the retina’s homeostasis and thermoregulation as well. The choroidal circulation is mainly controlled by autonomic nervous system, unlike the retina which is autoregulated but has no neural control [22].

**Fig. 1:**
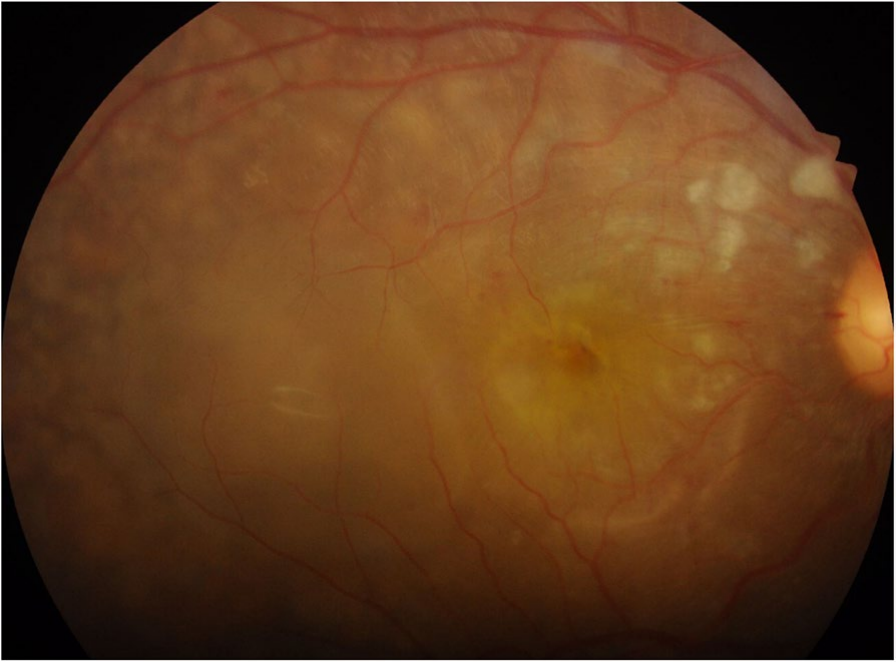
Fundus photograph of the right eye of a patient with hypertensive retinopathy showing cotton wool spots, serous detachment of the macula, and numerous deep pale yellow Elschnig spots surrounding the posterior pole

**Fig. 2:**
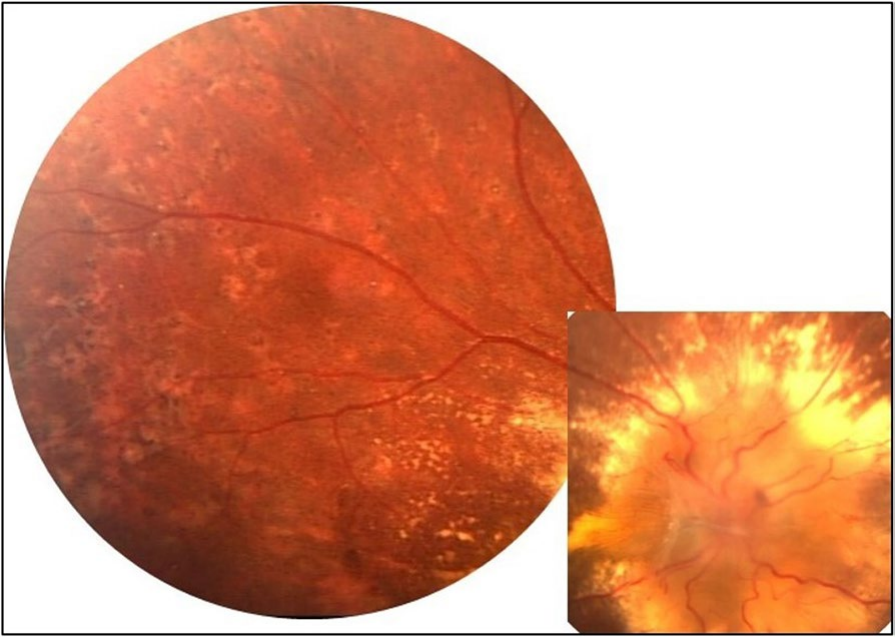
Montage color fundus photograph of the right eye of a patient with hypertensive retinopathy showing severe papilledema with exudation and pigmented Elschnig spots with atrophic halo in the peripheral retina (Courtesy of Prof. Mehmet Yasin Teke, Department of Ophthalmology, University of Health Sciences, Ulucanlar Eye Education and Research Hospital, Ankara, Turkey)

**Fig. 3:**
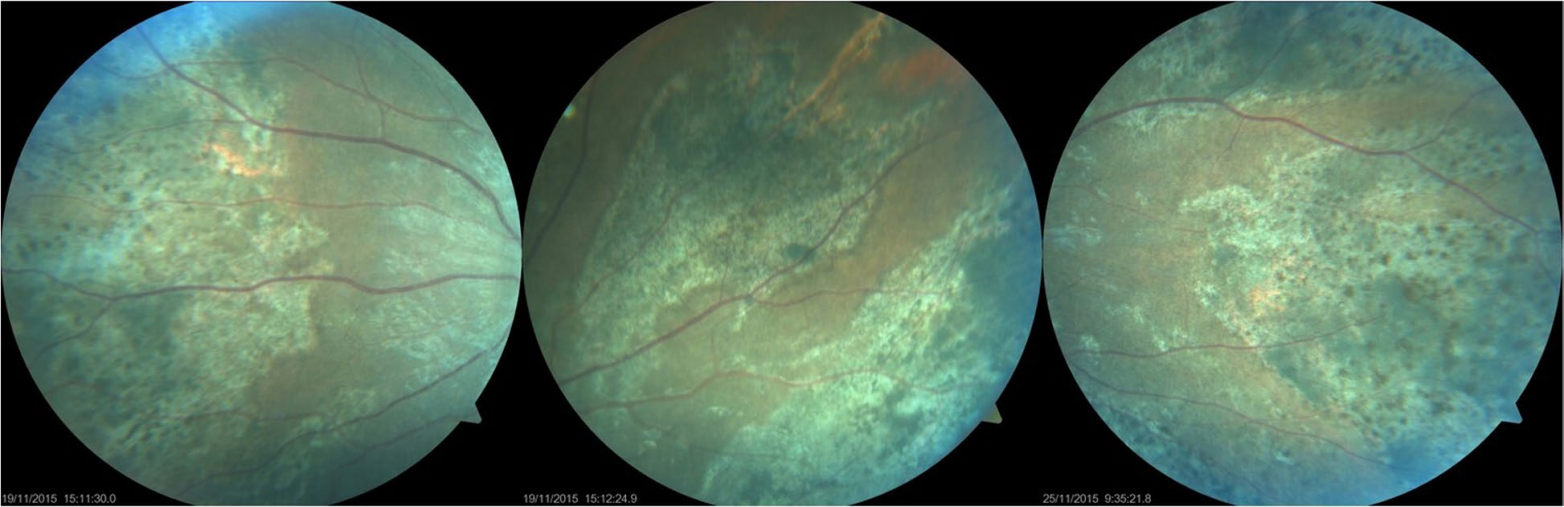
Color fundus photographs of a patient with hypertensive disease and renal insufficiency showing peripheral wedge-shaped triangular areas of pigmentary changes in both eyes indicating sequelae of the Amalric sign of choroidal infarction. (Courtesy of Prof. Gürsel Yilmaz, Baskent University Faculty of Medicine, Department of Ophthalmology, Ankara, Turkey)

Ophthalmoscopic signs of inflammatory or noninflammatory choroidal circulatory disturbances range from subtle changes in the fundus to readily visible lesions or even choroidal effusions and serous retinal detachments. The clinical findings of choroidal stromal inflammation have been well described in immune-mediated diseases such as birdshot retinochoroiditis and Vogt-Koyanagi-Harada (VKH) disease which primarily involve the choroid in a diffuse manner, or in secondary random involvement of the choroid, as seen in sarcoidosis [20]. Inflammatory ischemia of the choriocapillaris also has distinct clinical phenotypes such as acute posterior multifocal placoid pigment epitheliopathy (APMPPE) and serpiginous choroiditis [20]. Noninflammatory ischemic swelling of the choroid and impaired pump function of the retinal pigment epithelium (RPE) as a result of ischemic damage may mimic VKH disease with serous retinal detachments. Placoid lesions as in APMPPE may also occur when the choroid is involved in systemic vasculitides. Other signs of choroidal vasculopathy in systemic vasculitis may be indistinguishable from hypertensive choroidopathy which is mostly seen in younger patients with acutely elevated severe arterial hypertension [21]. Occlusion of small choroidal terminal arterioles is manifested as Elschnig spots, appearing as discreet deep retinal pale-yellow spots due to focal RPE infarction, particularly in the posterior pole (Fig. 1). Later they become irregularly pigmented with a depigmented halo (Fig. 2) [21, 23–25]. Occlusion of larger choroidal arterioles cause small geographic lesions. When the large choroidal vessels, such as the main trunks of the short posterior ciliary arteries or the branches of the long posterior ciliary arteries supplying the peripheral choroid are partially or completely occluded, the triangular sign of Amalric is seen, which is characterized by wedge-shaped triangular areas of chorioretinal opacification with their apex directed towards the macula or the optic disc [26, 27]. These triangular zones of choroidal, RPE, and outer retinal infarction later appear as atrophic areas with pigmentary changes (Fig. 3), typically less extensive in size than the area of apparent ischemia [21, 26]. Siegrist’s streaks are linear pigmentations overlying sclerosed choroidal vessels, first described in hypertensive choroidopathy [21, 28].

### Imaging of choroidal vasculopathy

Inflammatory diseases may affect both retinal and choroidal vasculature independently. As an example, necrotizing vasculitis in GPA or immune complex deposition and autoantibody formation in SLE can lead to inflammatory involvement of both retinal and choroidal vasculature which may result in choroidal infarction and retinal vascular occlusion. These vascular changes need dye-based angiography for detailed assessment [29].

#### Fluorescein angiography

This imaging modality has been the gold standard to evaluate retinal circulation. Even though early studies of choroidal circulation have been based on FA imaging, the limitations of FA for visualization of the choroid are manifold. Fluorescein usually appears in the choroidal circulation approximately 1 s before it appears in the retinal circulation. A patchy filling pattern is seen in normal individuals; however, choroidal ischemia may be implied if there are large areas with a perfusion delay of longer than 6 s. Because of the rapid leakage of fluorescein from fenestrated choriocapillaris, only a choroidal flush will appear on most angiograms. Information from the choriocapillaris can be obtained in the very early frames, and choriocapillaris filling defects may be visualized in up to about 60 s. Choroidal fluorescence is relatively blocked by intact RPE screen. On the other hand, FA is useful in detecting leakage from RPE and serous retinal detachments causing pooling of dye as well as sequelae such as RPE atrophy producing window defects. The early hypofluorescent and late hyperfluorescent pattern of choriocapillaropathies such as APMPPE is also useful in the diagnosis of these entities.

#### Indocyanine green angiography (ICGA)

This imaging modality has been the gold standard in the evaluation of choroidal circulation because the protein-bound ICG dye has limited diffusion through the fenestrations of the choriocapillaris and the spectral properties of ICG allow penetration of the RPE for visualization of the choroid [29]. In the early phase (up to 3 min), filling of both medium and large choroidal vessels are well visualized. In the middle phase (6–12 min), staining and leakage of individual choroidal vessels as well as diffuse fuzziness of the vessels become apparent in choroidal vasculitis. Choroidal granulomas appear as hypofluorescent dots and choriocapillaris nonfilling is seen as patches of hypofluorescence. In the late phase (> 20 min), large choroidal vessels appear dark against the ICG-impregnated choroidal stroma and choriocapillaris nonperfusion appears as even darker patches of hypofluorescence. Late diffuse zonal choroidal hyperfluorescence may result from abnormal diffusion of ICG from damaged choroidal vessel walls. Pinpoint spots of ICG hyperfluorescence seen in middle and late phase also indicate choroidal inflammation. ICGA has been especially useful in the detection of subclinical choroidal inflammation and ischemic choroidopathy.

#### Spectral domain optical coherence tomography (SD-OCT), enhanced depth imaging (EDI-OCT), and swept source (SS-OCT)

These imaging modalities allow noninvasive visualization of the retinal and choroidal morphology. While SD-OCT can provide good-quality images of the vitreous, the retina and the RPE and hence is useful in the detection and monitoring of serous retinal detachment and changes in the outer retinal layers secondary to choroidal inflammation, EDI-OCT, still based on SD technology, is used to visualize choroidal morphology. Highly pigmented eyes and thick choroids limit acquisition of good quality images by EDI-OCT. These limitations have been overcome by the development of SS-OCT, which provides high quality images of both retinal and deep choroidal images simultaneously [30]. In recent years, measurement of choroidal thickness (CT) with EDI-OCT or SS-OCT has been described as a choroidal imaging biomarker to identify subclinical inflammation and the risk of relapse in systemic autoimmune diseases [31, 32]. It has been shown that the CT increases in active periods of inflammatory diseases with vascular involvement and decreases with treatment. Moreover, recurrent inflammatory attacks and long-term disease can cause thinning and atrophy of the choroid because of the damage on microvasculature [32]. Choroidal vascularity index (CVI) analysis is also being used in order to assess the choroidal vascular morphology by binarization of OCT scans and the calculation of the proportion between the luminal and the total choroidal area. While the CT may be influenced by physiological variables such as axial length, refractive error, intraocular pressure, and systolic blood pressure, CVI is not affected by most of these variables and has been reported to be a relatively more stable marker of choroidal vascularity [30, 31, 33].

#### Optical coherence tomography angiography (OCTA)

It is a non-invasive dye-less imaging modality that has been introduced for depth-resolved imaging of the retinal and choroidal vasculature by detection of endoluminal flow [29]. OCTA provides qualitative and quantitative assessment of retinal vascular changes in retinal vasculitis, but is also helpful for the diagnosis and monitoring of several inflammatory and ischemic choroidal pathologies [30, 31, 33, 34].

**Fig. 4:**
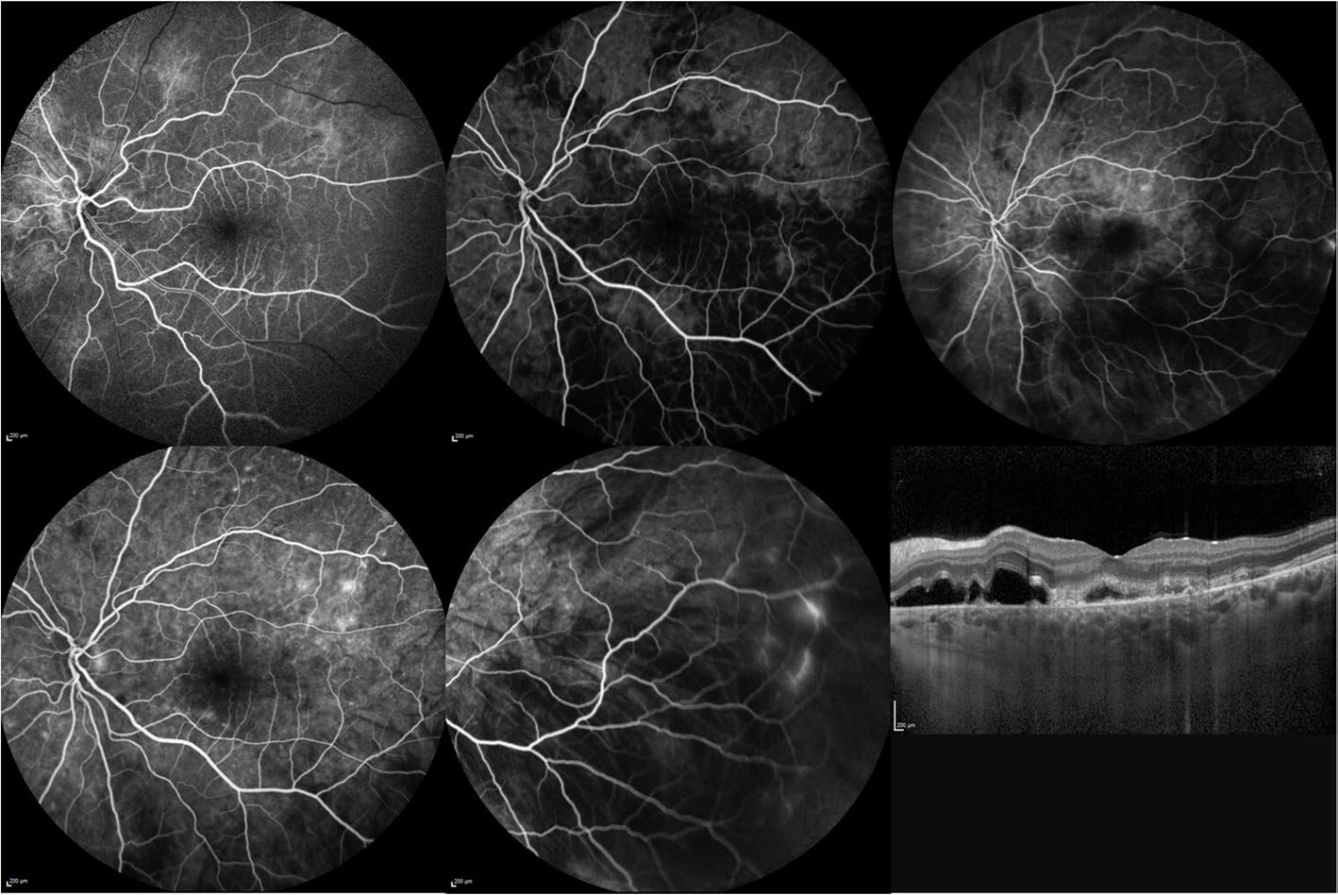
Fluorescein angiography and optical coherence tomography imaging of the left eye of a 20 year-old woman with Takayasu arteritis. Fluorescein angiography shows delayed patchy filling of the chororoid at 14 s (**top left**), 40 s (**top middle**), and 3 min (**top right**), pinpoint leaks at the level of retinal pigment epithelium seen at 3 min (**top right**) becoming more evident at 6 min (**bottom left**). Temporal peripheral frame at 6 min shows choroidal nonperfusion and focal leak of peripheral retinal vessels (**bottom middle**). SD-OCT scan of the macula shows serous retinal detachment (**bottom right**)

**Fig. 5:**
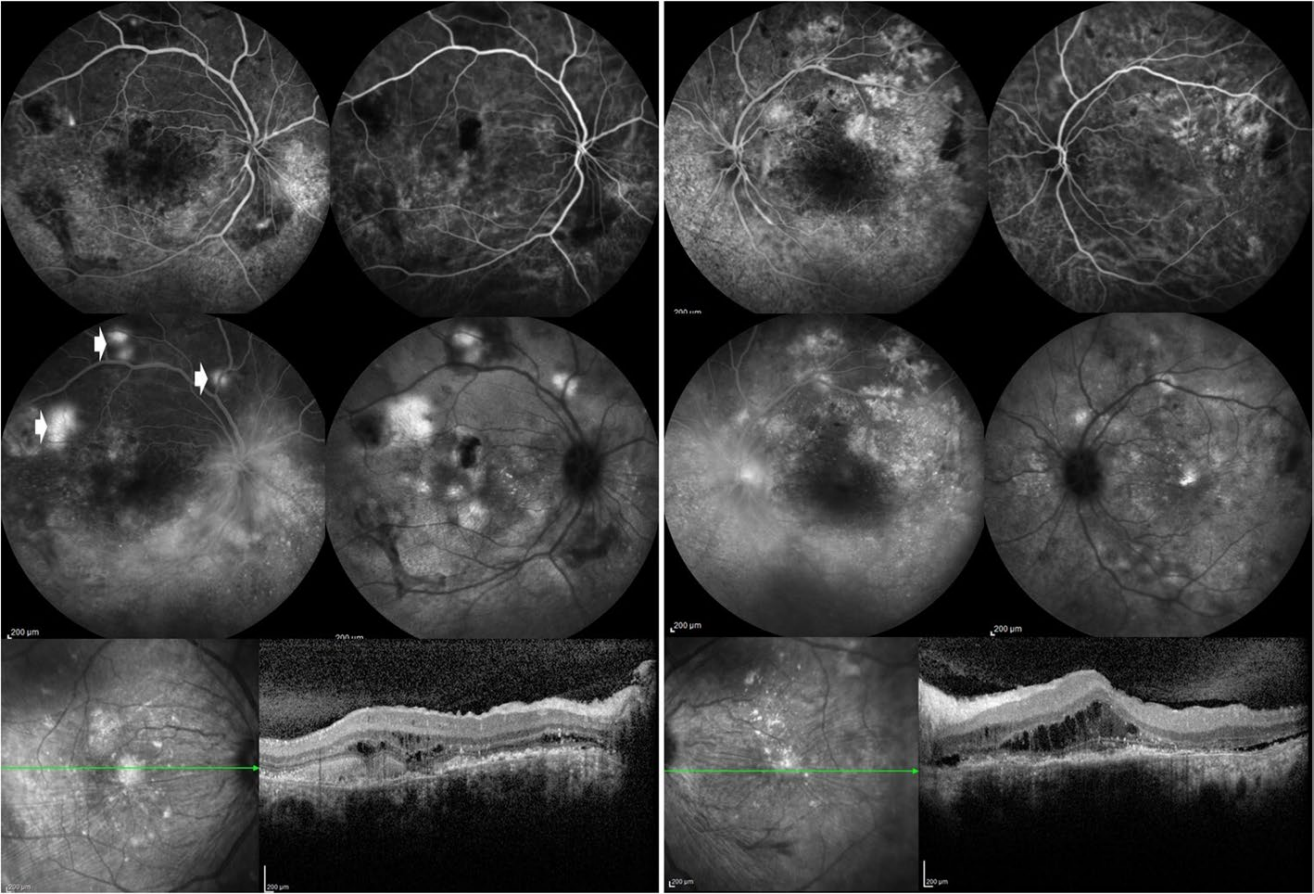
Dual fluorescein-indocyanine green angiography and optical coherence tomography imaging of a 36 year-old woman with systemic lupus erythematosus unresponsive to aggressive immunomodulatory treatment. Early phase fluorescein angiography (FA) shows extensive retinal pigment epithelial changes with window defects in the right (**top row, first frame**) and the left eye (**top row, third frame**); and late phase FA shows hyperfluorescence of the optic disc and peripapillary vasculature as well as subretinal staining/pooling (arrows) in the right eye (**middle row, first frame**) and staining of the optic disc and vessel walls in the left eye (**middle row, third frame**). Early phase indocyanine green angiography (ICGA) shows dilated and hyperfluorescent choroidal vessels in both the right (**top row, second frame**) and the left eye (**top row, fourth frame**); and late phase ICGA shows more numerous and larger hyperfluorescent areas than revealed by FA in the right eye (**middle row, second frame**) and also scattered hyperfluorescent spots in the left eye (**middle row, fourth frame**). SD-OCT shows extensive damage to the outer retina with chronic cystoid changes, subretinal hyperreflective material, and retinal pigment epithelium loss and irregularities in both the right (**bottom row, left**) and the left eye (**bottom row, right**)

**Fig. 6:**
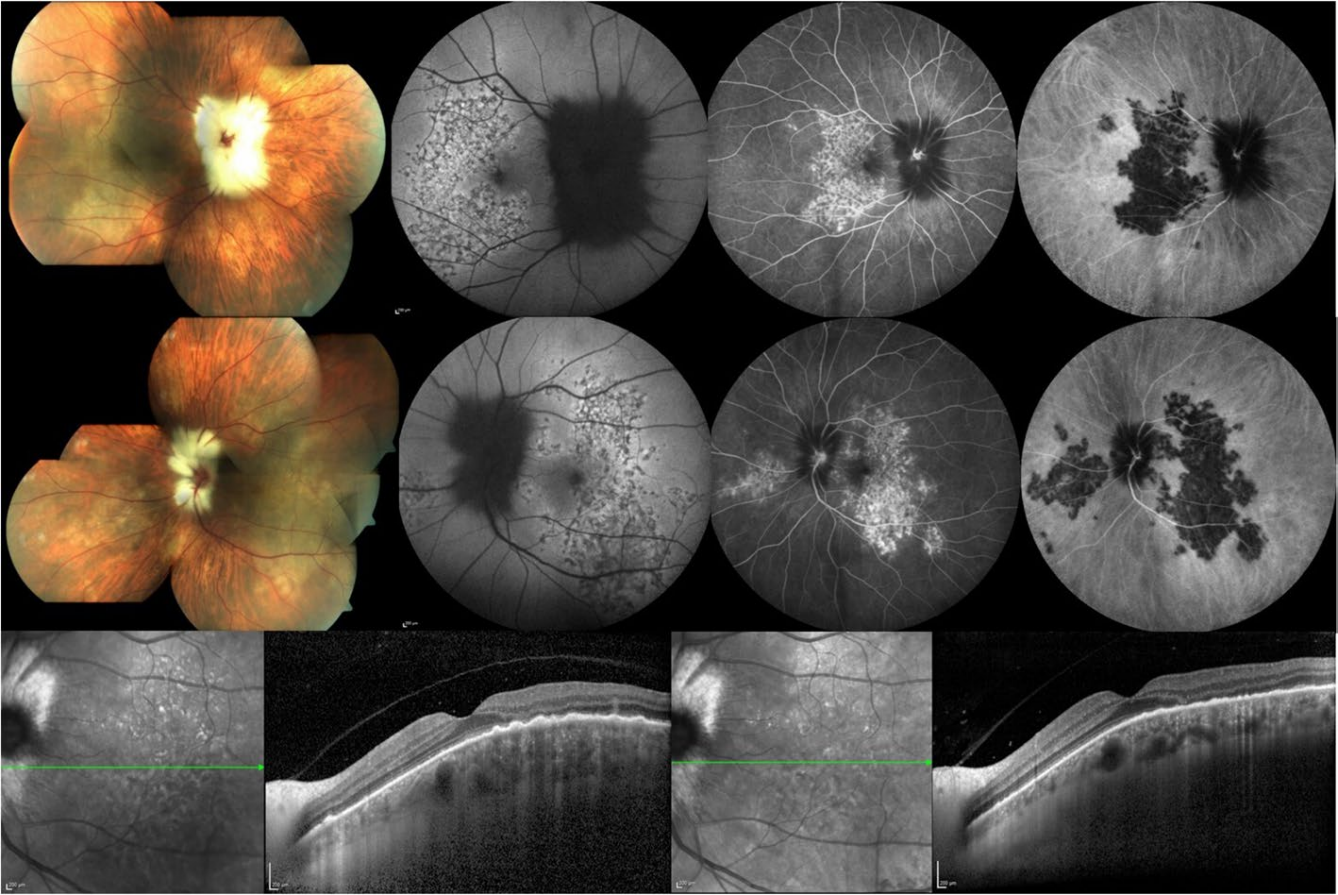
Color fundus photography, dual fluorescein-indocyanine green angiography and optical coherence tomography imaging of a 63 year-old woman who presented with photopsias and loss of vision after tapering oral corticosteroid given in combination with azathioprine for the treatment of granulomatosis with polyangiitis diagnosed 2 months prior. Montage color fundus photographs of the right (**top row, first frame**) and the left (**middle row first frame**) eye show myelinated nerve fibers obscuring the optic disc margins, deep creamy-white serpiginoid choroiditis involving the posterior pole in both eyes and inferonasal to the disc in the left eye. Fundus autofluorescence imaging shows a granular pattern of hyper- and hypoautofluorescence of the lesions in the right (**top row, second frame**) and the left eye (**middle row, second frame**). Dual fluorescein-indocyanine green angiography frames at 8 min show window defects and staining on fluorescein angiography and diffuse hypofluorescence of the lesions on indocyanine green angiography in the right (**top row, third and fourth frames, respectively**) and the left eye (**middle row, third and fourth frames, respectively**). EDI-OCT of the left eye shows loss of outer retinal layers, RPE irregularities, thickening of the choroid, and hyperreflective dots in the choroid in the involved area (**bottom row, left**). After intravenous pulse methylprednisolone 1 g for 3 days, EDI-OCT shows partial improvement of RPE irregularity and reduced thickening of the choroid (**bottom row, right**). Visual acuity increased from counting fingers in both eyes to 20/40 in the right and 20/32 in the left eye. Active and latent tuberculosis had already been ruled out and the patient subsequently received cyclophosphamide therapy

Multimodal imaging of the choroid using above mentioned modalities helps early detection of choroidal involvement which may have prognostic implications in systemic vasculitic diseases. Multimodal imaging has also improved our understanding of the pathogenesis and evolution of long known clinical signs of choroidal ischemia [23–28]. Figure 4 shows choroidal ischemia in a patient with Takayasu arteritis; Fig. 5 shows multimodal imaging in a patient with advanced lupus choroidopathy; and Fig. 6 shows multimodal imaging of serpiginoid choroiditis in a patient with GPA.

The following section provides a compilation of literature on choroidal involvement in selected systemic vasculitic diseases.

1. **Large vessel vasculitis (LVV)**
  *Takayasu arteritis (TAK)* is an inflammatory vascular disease which mostly affects young women and may cause ocular manifestations including Takayasu retinopathy (15%), ocular ischemic syndrome (7%), and hypertensive retinopathy (16%) [35]. The study by Chun et al. evaluating 78 patients with TAK showed that the arm-to-retina circulation time was delayed in all cases, while retinal arteriovenous filling time was only delayed in moderate to severe cases. They also reported delayed choroidal filling, vascular dilation, microaneurysms, vessel staining, ischemic areas, arteriovenous shunts and retinal neovascularization as other angiographic findings [36]. Although choroidal ischemia has been cited among its ocular manifestations [37], there is no specific study of choroidal involvement of TAK in the literature yet.
  *Giant cell arteritis (GCA)* also known as temporal arteritis is a chronic granulomatous vasculitis of the large and medium- sized arteries affecting people over 50 years of age and may cause irreversible visual loss [1, 38]. It is known for a long time that the disease can be accompanied by various ocular clinical and FA findings [39, 40]. In a cohort including 56 patients with GCA, 19 (33.9%) patients were detected to have ocular manifestations [38]. Arteritic anterior and posterior ischemic optic neuropathy was the most common manifestation (63.2%), followed by cotton wool spots (15.8%), central retinal artery occlusion (10.5%) and multifocal choroidal ischemia (5.3%). Patient with choroidal ischemia presented with pigment epithelial changes in macular area and choroidal filling defects [38]. Quillen et al. stated that the choroidal non perfusion accompanies occlusion of the ophthalmic artery or its branches in most of the time but may also be the only ocular manifestation of the disease [41]. GCA cases presenting with unilateral or bilateral choroidal infarction leading to the appearance of Amalric sign have been reported [41–43]. A case of GCA presenting with submacular choroidal ischemia without change in dye-filling pattern of retinal vessels has also been reported by Olali et al. [44]. Macular ischemia resulted with pigment epithelial changes and loss of foveal architecture [44]. Gandhi confirmed that GCA can cause localized ischemic patches in the choroid while sparing the optic nerve and retinal arteries and recommended to consider the disease in inexplicable visual loss of older patients [45]. More recently, Ness et al. reported a 64-year-old man who was referred with bilateral painless visual loss in whom they noticed placoid maculopathy related to choroidal hypoperfusion. In this patient who was then diagnosed as GCA, FA showed delayed filling of the macular choroid and late staining of the placoid lesion, whereas ICGA showed hypofluorescence of the lesion throughout the test. They proposed that temporal short posterior ciliary arteritis resulted in ischemia of the subfoveal choriocapillaris and subsequent damage to the overlying RPE and outer retina in a placoid pattern [46]. There are also reports of central serous chorioretinopathy (CSCR) during systemic corticosteroid treatment in patients with GCA [47–49]. Thus, CSCR should be considered in the differential diagnosis of vision loss in GCA patients who are on systemic corticosteroid treatment, especially if there is no other evidence for disease activity. Because increasing the corticosteroid dose might worsen the visual outcome, it is important to differentiate CSCR from ischemic choroidopathy by performing a complete ophthalmological examination including multimodal imaging.
2. **Medium vessel vasculitis (MVV)**
  *Polyarteritis nodosa (PAN)* is a rare multisystemic necrotizing vasculitis characterized by patchy but widespread involvement of small to medium sized arteries and can affect almost any organ. Depending on which vessels are affected, PAN can involve every tissue of the eye. Ocular manifestations have been reported in 10–20% of cases and may be the presenting manifestation of this life-threatening disease. Episcleritis, scleritis, peripheral ulcerative keratitis, neuro-ophthalmic manifestations including papilledema, cranial nerve palsies and diplopia, non-granulomatous uveitis, cotton-wool spots, retinal vasculitis, retinal arterial occlusions, and orbital involvement may occur [50, 51]. In 1946, Goldsmith published a patient with PAN who presented first with retinal arterial and choroidal involvement. Postmortem examination of this patient revealed edematous and hyalinized ciliary processes, serofibrinous and hemorrhagic detachment of the ciliary body and peripheral choroid, and focal areas of lymphocytic and mononuclear cell infiltration around small arteries in the posterior choroid [52]. Normal retinal circulation with delayed choroidal filling and staining of the involved arterial segments showing arteritis have been emphasized as characteristic FA findings in PAN patients [51]. Involvement of posterior ciliary arteries and choroidal vessels resulting with choroidal infarcts and exudative retinal detachments, and choroidal vasculitis have been reported to be frequent [51]. Hsu et al. described a patient with PAN who presented with bilateral choroidal infarction showing an Amalric sign, along with central retinal artery occlusion in one eye, and anterior ischemic optic neuropathy in the other [53]. Ramtohul et al. reported segmental choroidal vasculitis and nodular posterior scleritis in a patient already diagnosed as PAN [54]. In mid-phase and late-phase ICGA, segmental choroidal vessel staining and late leakage without choroidal ischemia have been demonstrated. OCT scan through this choroidal vasculitis area showed hyperreflective thickening of the inflamed choroidal vessel walls and chorioretinal folds, which resolved after pulse steroid therapy [54]. Another presentation of choroidal involvement is the development of APMPPE which has been reported in two cases, one of them having mixed features of GPA and PAN [55, 56]. In a study evaluating choroidal changes using EDI-OCT in children with PAN and PAN-like vasculitides, CT has been found to increase compared to healthy controls [57]. CVI however, did not show any significant difference between the groups. This increase in CT could not be correlated with systemic inflammatory findings [57].
  *Kawasaki disease (KD)* is a childhood systemic vasculitis which is the most prevalent acquired cause of heart diseases. The most common ocular manifestations are bilateral non-exudative conjunctivitis (64%) followed by uveitis (36%) which is mostly anterior [58]. Superficial punctate keratitis, vitreous opacities, papilledema, and subconjunctival hemorrhage are other less frequent ocular findings [59]. Choroidal involvement has not been reported so far.
3. **Small vessel vasculitis (SVV)**
  *Antineutrophil cytoplasmic antibody –associated vasculitis (AAV)* is a group of autoimmune, multisystemic and potentially life-threatening diseases characterized by necrotizing vasculitis affecting small to medium sized vessels. It comprises three distinct disease subtypes: GPA (formerly Wegener’s granulomatosis), MPA, and EGPA (formerly Churg-Strauss syndrome). Upper and lower respiratory tracts and kidneys are the most commonly affected organs and systems [60]. Ocular involvement has been reported between 16% to 20% of AAV patients [5, 9, 61]. Among the subtypes of AAV; ocular involvement is most common in GPA followed by EGPA and MPA [60]. In GPA patients, ocular manifestations are present in almost half of patients and may be the initial features of the disease. Although orbital disease and scleritis have been reported to be more common, conjunctivitis, peripheral ulcerative keratitis, uveitis, optic neuritis and retinal vasculitis may also occur, with a wide range of severity [4, 8, 62].
    Choroidal involvement appears to be a rare manifestation of AAV. It has not been reported neither in a cohort of 63 GPA patients nor in a recently reported series of 18 AAV cases [61, 63]. However, there are many case reports describing different forms of choroidal involvement. Kinyoun et al. reported a choroidal involvement in two cases with systemic necrotizing vasculitis including one with GPA and the other with lymphomatoid granulomatosis [64]. This involvement has been described as a presumed choriocapillaritis induced ischemia and infarction of choriocapillaris-RPE-outer retina complex which could be treated with systemic corticosteroids and immunosuppressive agents [64]. Later, a few cases of APMPPE associated with GPA have been reported in the literature [55, 65]. FA and ICGA findings shown in Chiquet et al.’s report confirmed the occurrence of choriocapillaris ischemia which has been attributed to the inflammatory occlusion of choriocapillaris [65]. Damato et al. described a case of GPA who presented with bilateral serous retinal detachment showing marked choroidal ischemia affecting the whole posterior pole on ICGA imaging [66]. Choroidal vasculitis and ischemia improved significantly with plasmapheresis [66]. Mirza et al. and then Iida et al. published two different cases of GPA who presented with retinal arterial occlusion and bilateral choroidal infarcts [67, 68]. ICGA performed in the latter report showed a widespread filling defect of choroidal arteries and choriocapillaris [68]. In a post-mortem evaluation of posterior choroid in a 71-year-old man with GPA who died because of severe systemic complications of the disease, many foci of granulomatous inflammation similar to those seen in other organ involvements have been observed [69]. These foci consisted mostly of the epithelioid cells accompanied by lymphocytes and a few multinucleated giant cells. Rare minute foci of fibrinous necrosis in the choroid just beneath the choriocapillaris were observed. The choriocapillaris was also infiltrated by inflammatory cells and the capillaries were either stenotic or occluded. The choroidal vessels were surrounded by the dense inflammatory infiltrates. All these findings suggested a granulomatous choroiditis [69]. Lim et al. reported two cases of GPA who developed severe macular retinal vasculitis and choroiditis that healed with fibrosis, despite aggressive immunomodulatory therapy and good control of systemic disease [70]. Another rare type of choroidal involvement has been reported by Janknecht et al. [71]. The authors described a case of GPA who presented with sclerochoroidal granuloma simulating uveal melanoma. They could not differentiate uveal melanoma on ocular ultrasound, but histological examination showed a chronic necrotizing granulomatous inflammation [71]. In association with chorioretinal granulomas or posterior scleritis; choroidal folds, choroidal effusions, or an exudative retinal detachment may also be seen [62]. Few cases of AAV associated necrotizing scleritis which developed combined choroidal and retinal detachment, sometimes mimicking a choroidal tumor have been reported [72, 73]. Pulido et al. reported ocular manifestations of six patients with AAV, in whom one with GPA had bilateral choroidal folds. Ocular ultrasound revealed bilaterally thickened choroidal layers which appeared to be infiltrative [74].
    Dry eyes and epiphora are the most common ocular manifestations of EGPA. Although rare, retinal arterial occlusions and extraocular manifestations including vascular occlusions of muscles and orbit vessels can also be seen [75]. Similarly, rare cases of EGPA presenting with multifocal choroidal infarcts have been reported [76–78].
    Ocular involvement in MPA is very rare including scleritis, ischemic optic neuropathy, peripheral keratitis, uveitis, retinal cotton wool spots and APMPPE. Choroidal involvement other than APMPPE has not been reported [75, 79].
  *Immune-complex SVV* is characterized by vessel wall deposits of immunoglobulin and/or complement and small vessels are predominantly affected. Glomerulonephritis is frequent but arterial involvement is much less common compared to AAV [3]. *Anti–glomerular basement membrane (anti-GBM) disease*, previously known as Goodpasture’s syndrome may cause ocular manifestations which are not specific for the disease. Anti-GBM antibody was shown to be bounded to the ciliary epithelium and Bruch’s membrane in two cases with serous retinal detachment [80]. While fibrinoid occlusion of the choriocapillaris and necrosis of the RPE, together with choroidal hemorrhage have been shown in light microscopy, electron microscopy revealed ultrastructural changes in basement membranes. Although not proven with biopsy, a case with bilateral choroidal neovascular membrane associated with anti-GBM disease has been reported [81]. *Cryoglobulinemic vasculitis (CV)* is characterized by cryoglobulin immune deposits affecting small vessels and associated with cryoglobulins in the serum. This type of vasculitis affects mostly the skin, glomeruli, and peripheral nerves and may be idiopathic or secondary to other etiologies [3]. Although rare, cryoglobulinemia associated ocular findings such as retinal vascular tortuosity and occlusions, cotton wool spots, retinal hemorrhages and uveitis have been described. Few cases of CSCR have also been reported [82]. This may be attributed to the corticosteroid use in some of the cases or to the increased capillary permeability caused by excessive plasma protein concentration resulting with accumulation of subretinal and sub-RPE fluid [82]. An extensive triangular wedge-shaped choroidal infarction leading to irreversible visual loss has also been reported in a postpartum female with mixed essential cryoglobulinemia [83]. Loss of capillary layers and dilated large vessel layer observed in the right eye of this case has been considered as a sign of previous acute choroidal injury [83]. The most plausible hypothesis explaining the development of ocular findings includes ischemia secondary to paraproteinemia-related hyperviscosity, cryoglobulin induced inflammation and occlusive process caused by the unusual temperature-dependent globulin solubility [83]. In *IgA vasculitis* (Henoch-Schönlein) characterized by abnormal IgA deposits affecting small vessels, rare cases of ocular involvement including central retinal artery or vein occlusion, anterior ischemic optic neuropathy, ischemic retinal vasculitis, scleritis and sclerokeratitis have been reported [84–86]. Choroidal involvement has not been reported either in IgA vasculitis or in hypocomplementemic urticarial vasculitis.
4. **Variable vessel vasculitis (VVV)**
  In this subtype of systemic vasculitis, there is no predominant size or type of vessel involvement. Vessels of any size (small, medium, and large) and any type (arteries, veins, and capillaries) can be affected, Behcet’s disease (BD) and Cogan’s syndrome being the most important two examples. Because of the frequency of vasculitis, BD and Cogan’s syndrome are considered as primary vasculitis rather than vasculitis associated with a systemic disease [3].
  *Behçet’s disease (BD)* is a multi-system inflammatory vasculitis of unknown etiology. Ocular involvement, which is one of the major manifestations of the disease, is characterized by occlusive and leaky retinal vasculitis affecting mostly the retinal veins. Diffuse retinal capillary leakage is the most common FA finding that is frequently observed even during clinically quiescent periods.
    A prominent local or diffuse infiltration of neutrophils into the choroid during an acute attack and fibrosis of the choroid during the chronic phase of BD have been shown previously [87]. On the other hand, in an expert-guided algorithm proposed by Tugal-Tutkun et al., the presence of choroiditis has been found to be one of the ocular findings decreasing the probability of the diagnosis of BD uveitis [88]. Thus, the choroidal involvement is not an expected manifestation of BD uveitis. The absence of occult choroiditis may need to be confirmed by multimodal imaging [89]. Although signs of choroidal vasculitis have been shown on ICGA, dark dots representing choroidal granulomas are never seen in BD uveitis [89]. Several studies evaluated ICGA findings of BD uveitis. These findings include delayed and irregular filling of choriocapillaris, choroidal fuzziness, staining of choroidal vascular walls, hyperfluorescence of choroidal stromal vessels, hyperfluorescent spots and hypofluorescent plaques [90–94]. These findings are nonspecific and have been found to not correlate with FA findings. They reflect the immunological processes of edema, fibrosis and leukocyte infiltration in the stroma and vascular walls [90]. ICGA findings do not provide additional information about disease activity, evaluation of disease progression or treatment monitoring [89, 94].
    Several studies have also investigated the CT of BD patients by using EDI-OCT [95–104]. These studies revealed conflicting results which may be attributed to the non-homogenous patient populations in terms of activity, anatomical involvement and duration of uveitis. Some of them reported an increase of CT in the active phase of the disease which decreased by the resolution of the inflammation [95–97]. While Kim et al. [95] found a significant association between CT and retinal vascular leakage in FA, Ishikawa et al. [96] found a significant correlation between CT and clinical ocular inflammation scores. A recent study investigating the relationship between CT and FA leakage scores demonstrated that both were significantly higher in the active phase compared to convalescent phase [97]. The authors showed that only the FA leakage from the macula, but not the peripheral retina or the optic disc were significantly correlated with the corresponding changes in CT. They suggested CT measurement as a non-invasive method for evaluating inflammation near the macula in BD uveitis [97]. There are also studies that have shown an increased CT compared to healthy controls in Behçet patients, not only in acute attack but also during remission period [95, 98, 99]. In contrast, Coşkun et al. reported that the choroid was thinner in eyes with BD uveitis and that there was no significant difference in the CT between eyes with an acute attack and those in remission [100]. This thinning has been attributed to the longer disease duration of uveitis which can provoke atrophy and fibrosis in the choroid [100]. Consistent with the results of this study, Park et al. demonstrated a significant long-term thinning of the choroid over time in BD patients with posterior segment involvement; and the decrease in CT has been shown to associate with length of active inflammation [101]. Similarly, Yesilirmak et al. demonstrated an increase of CT in the active phase of BD uveitis and thinning in end-stage disease [99]. In a small retrospective study, Mittal et al. compared CT of non-ocular BD patients and healthy control subjects and reported a significantly thinner CT in BD suggesting subclinical manifestations in the choroid [102]. In a study by Chung et al. investigating whether subfoveal CT is an indicator of subclinical ocular or systemic inflammation, eyes with non-ocular BD, eyes with previous history of BD uveitis in an inactive period and healthy controls were evaluated. Subfoveal CT and disease activity score were significantly higher in non-ocular BD group [103]. In contrast to Mittal et al. who suggested choroidal thinning as a manifestation of subclinical choroidal involvement [102], Chung et al. stated that the increased CT may be a clinical indicator of subclinical ocular and systemic inflammation in BD patients without active ocular inflammation [103]. Supporting this data, in an OCTA study, Goker et al. demonstrated that flow area of the choriocapillaris layer at 1 mm, 2 mm, and 3 mm radius areas was higher in the non-ocular BD group than in healthy control group and speculated that patients with non-ocular BD may have subclinical choroidal involvement [105]. On the other hand, Onal et al. did not observe any significant increase in subfoveal CT during an acute uveitis attack in Behçet patients compared to healthy control subjects [104]. However, they showed a choroidal stromal expansion which was not associated with thickening of the choroid in eyes with active BD uveitis. An increased choroidal stroma to choroidal vessel lumen ratio was observed in CVI analysis in this study, suggesting a predominant involvement of inner choroidal vessels [104].
  *Cogan’s syndrome vasculitis* is characterized by ocular inflammatory lesions including interstitial keratitis, uveitis, and episcleritis, and inner ear disease, including sensorineural hearing loss and vestibular dysfunction. Vasculitic manifestations may include arteritis of all sizes. The main site of ocular vascular involvement is the small vessels of the vascularized layers of the anterior globe [3]. Posterior segment involvement is extremely rare. There is only one reported case of atypical Cogan’s syndrome with serous retinal detachment which is associated with choroidal inflammation [106].
5. **Vasculitis associated with systemic disease**
  This category includes vasculitis which is secondary to systemic disease such as SLE, rheumatoid arthritis, systemic sclerosis and relapsing polychondritis.
  *Lupus vasculitis* may affect several organs and systems in the body, often including small vessels of the eye. The most common ocular manifestations of SLE consist of keratoconjunctivitis sicca, optic nerve changes, and retinal and choroidal vasculitis [32]. In a recent series, among 161 patients with SLE, 50 patients (31.1%) had at least one ocular manifestation. Lupus choroidopathy was the least common presentation and seen in only one patient (0.6%) [107]. Although the pathogenesis of lupus choroidopathy remains uncertain, mechanisms such as immune complex deposition in the choroid, autoantibodies against RPE and thrombotic microangiopathy leading to hypoperfusion of the choroid, RPE damage, and leakage into the subretinal space have been considered [108]. Fibrin deposition, which often occurs in the vessel wall and lumen may damage the choroidal vasculature [109]. Choroidal infiltration with inflammatory cells, immunoglobulin and complement deposition in the choroidal vasculature, and damage to the RPE have all been shown in histopathological studies [110]. Hypertension which is frequent in patients with nephropathy may also play a role in lupus choroidopathy by causing choroidal ischemia due to choroidal vascular occlusion and subsequent serous retinal detachments [108]. Lupus choroidopathy presenting with serous retinal and pigment epithelial detachment occurs especially in severe cases of SLE. All cases of choroidopathy reported by Nguyen et al. [108] and Jabs et al. [111] had evidence of severe systemic vascular disease. Nguyen et al. suggested that, in an active systemic disease associated with severe nephropathy or central nervous system vasculitis, choroidal involvement should be suspected and vice versa. They emphasized that lupus choroidopathy serves as a sensitive indicator of disease activity [108].
    Depending on the level of inflammation and the time of evolution, CT may change in SLE patients. Increased CT may reveal preclinical choroidopathy, neuropathy and nephropathy [32]. In a recent study by Braga et al., patients with lupus nephritis had significantly thicker choroid compared to SLE patients without nephritis and healthy controls [112]. Thickening was more significant at the inferior and nasal parts of the macula which were the thinner areas of the control choroids. These results suggested an increased risk of choroidopathy in patients with lupus nephritis. There was no relation between choroidal thickening and disease duration and the authors suggested that this thickening might be correlated with histopathological changes like those occurring in kidney glomeruli [112]. In another study, Lee et al. reported that the CT did not differ between patients with lupus nephritis in complete renal remission and SLE patients without nephritis [113]. Unlike many previous studies, in a large series by Dias-Santos et al., a thinning of the choroid in SLE patients has been reported, particularly in those with nephritis and taking anticoagulants [114]. This is the only study reporting a thinner choroid in patients with lupus nephritis which was explained by the compromised choroidal blood supply and chronic ischemia [114].
    As previously mentioned, lupus choroidopathy may be a precursor of systemic activation. Detection of the choroidopathy or preceding increased CT may be an opportunity for early diagnosis of subclinical systemic activation and prevention of irreversible damage [108, 112, 115]. Ferreira et al. compared 43 SLE patients without ophthalmologic symptoms and 80 healthy controls and observed a thicker choroid in SLE patients [116]. In contrast, a study comparing 58 patients with inactive SLE with 58 healthy controls, revealed a significantly thinner choroid in SLE patients [117]. This thinning has been attributed to chronic vasculitis, immune and complement deposition in vascular endothelium of the choroid causing a reduction in choroidal blood supply or chronic ischemia leading to choroidal atrophy. Inclusion of patients who were in inactive phase of the disease may have also contributed to this result [117]. An important issue is to differentiate lupus choroidopathy needing high-dose corticosteroid treatment from CSCR which may also occur in SLE patients [118, 119]. Direct RPE damage due to the ischemic process in the choroid, long-term immunosuppressive treatment including corticosteroids, and systemic hypertension associated with nephritis are the factors playing a role in the pathogenesis of CSCR in SLE patients [117–119]. History of systemic disease preceding the choroidal involvement in most of the cases and multimodal imaging would help recognizing lupus choroidopathy. ICGA shows choroidal vascular abnormalities not evident on clinical examination. These include focal, transient hypofluorescent areas in the early phase showing filling defect, fuzziness of large choroidal vessels with late diffuse zonal hyperfluorescence indicating choroidal vascular leakage, wedge-like shaped choroidal hypoperfusion area, focal pinpoint spots of choroidal hyperfluorescence representing immune deposits at the choroid and Bruch’s membrane [118, 120]. Baglio et al. demonstrated drusen-like deposits with ICGA in all patients with renal disease. These deposits were not detectable by clinical and FA examination in most of the patients. Thus, they suggested the use of ICGA as an indicator of ocular involvement which could help in the decision to perform a renal biopsy [121]. FA shows delayed choroidal perfusion, choroidal non-perfusion areas, numerous pinpoint leakage areas with pooling corresponding to serous detachment and optic disc hyperfluorescence [108, 118]. OCT shows multiple serous retinal elevations, RPE changes and hyper-reflective dots. OCT is a useful tool not only for diagnosing but also for monitoring patients with choroidopathy [118, 122]. Invernizzi et al. described drusen-like deposits best detected in SD-OCT in patients with SLE [123]. These deposits were found to be independent of renal disease suggesting complement alteration as a primary cause of the lesions. However, patients without a renal disease showed smaller and less numerous deposits. The presence of these drusen-like deposits has been attributed to the anatomic similarities between the glomerulus and the choriocapillaris/Bruch membrane/RPE complex [123].
    It has been postulated that aggressive control of both the systemic disease activation and associated hypertension are important in the treatment of choroidopathy [111]. Focal laser photocoagulation and/or photodynamic therapy may have an adjunctive effect in resolving subretinal fluid especially in cases with insufficient response to systemic therapy [124].
    Another rare presentation of SLE is the choroidal detachment which may be severe and occurring bilaterally. Severe choroidal detachment was presumably caused by the decrease in plasma oncotic pressure due to prominent hypoalbuminemia inducing a leakage of plasma components from the choroidal vessels [125]. Choroidal effusion may rarely cause an anterior shift of the lens–iris diaphragm leading to secondary angle closure glaucoma and may be the presenting sign of SLE [126]. Few cases with choroidal neovascularization and polypoidal choroidal vasculopathy secondary to SLE have also been reported [127]. Another rare presentation of choroidal involvement is the punctate inner choroidopathy which has been reported in a single case of SLE [128].
  *Rheumatoid arthritis (RA)* is a multisystem autoimmune disease affecting synovial-lined joints in the hands, feet, wrists and knees. Various tissues such as cartilage, bone, ligaments, tendons and blood vessels become destructed. Vasculitis involves blood vessels of all sizes, particularly the small vessels and is associated with extra-articular involvements including the lungs, kidneys, cardiovascular system and the eyes. Ocular involvement occurs in 18% of patients, dry eye, episcleritis, scleritis, peripheral ulcerative keratitis being the most common manifestations [129, 130]. Due to vascular changes, rare cases with choroidal and retinal vascular involvement have also been reported [130].
    Most of the studies evaluating CT in RA patients revealed a thinner choroid in RA patients compared to healthy controls [116, 130–132]. It has been postulated that the possible reason for this thinning was the vascular involvement causing chronic vascular damage in the choroid. Increased levels of proinflammatory cytokines, especially endothelin-1 (ET-1) which is a potent physiological vasoconstrictor can also lead to choroidal vasculature narrowing, reduced blood supply and consequent choroidal thinning [130, 131]. Duru et al. did not find a significant difference in CT between patients with active RA and those in remission [130]. Furthermore, CT values were similar among mild, moderate and severe activity subgroups. They postulated that even when the disease goes into remission, choroidal thinning does not improve due to permanent damage of the choroidal vessels. There was no information about the patients’ treatment status [130]. On the other hand, in the study of Karti et al., although CT was decreased in all, patients on hydroxychloroquine (HCQ) treatment for 1 year had significantly thicker choroid when compared to the pre-treatment period [131]. This result has been explained by a decline in ET-1 levels and reduced ET-1 related vasoconstriction provided by HCQ treatment and considered as clinically negligible [131]. In contrast to the Duru et al.’s study [130], Kurt et al. [132] reported a significantly thinner choroid in patients with active disease than patients in remission. However, consistent with Duru et al.’s results, a relationship between activity level and CT has not been shown [132]. Contrary to these studies reporting a thinner choroid in RA patients, there is one study showing no difference [133] and another stating a significantly increased CT which was positively correlated with rheumatoid factor [134]. These conflicting results have been attributed to the heterogenous data about the treatment status of patients who were included in the above-mentioned studies [32].
    Another presentation of choroidal involvement was geographic choroiditis associated with systemic vasculitis due to RA. In the case reported by Matsuo et al., ICGA showed obstruction of both the choriocapillaris and choroidal arteries [135]. Also, a historical case with bilateral choroidal lesions considered as rheumatoid nodules and associated retinal detachment represents a rare manifestation of choroidal involvement in RA [136].
  *Systemic sclerosis (SSc)* is an idiopathic, chronic, multisystem autoimmune disease characterized by immune activation, vasculopathy and fibrosis. Small arteries and capillaries are affected leading to vascular occlusion and reduced capillary density [32]. Along with prominent vascular changes, ET-1 and angiotensin-2, which are potent vasoconstrictors have been found to increase and playing a role in the vasospastic activity. The choroid is also affected by these circulating mediators leading to impaired choroidal autoregulation and reduced blood flow [137]. Involvement of choroidal vessels with endothelial cell damage, basement membrane thickening, absence of pericytes, and abnormal material deposition of endothelium have been shown in postmortem histopathological studies [138]. In vivo studies using FA showed choroidal hypoperfusion, patchy non-perfusion areas and RPE atrophy occurring as a result of underlying vascular damage at the choroidal layer [139, 140]. Abdellatief et al. reported ICGA finding of choroidal vessel leakage in both eyes of a patient with SSc [141]. While Aydın et al. [142] did not find a significant difference in CT of SSc patients, several other studies showed a significantly thinner choroid in patients with SSc than in healthy controls [137, 143–146]. Ingegnoli et al. have reported a decrease in choroidal and macular thickness, as well as in ganglion cell complex in patients with primary Raynaud’s phenomenon (RP) [143]. This thinning was found to be more severe and extended in RP secondary to suspected and overt SSc. They suggested an early involvement of ocular microcirculation with significant reduction of choroidal perfusion [143]. Any significant difference has not been found regarding CT profile between patients with limited SSc and those with diffuse type of SSc [137, 144]. Moreover, duration of disease and treatment with calcium channel blocker were not found to correlate with CT change [137]. In an OCTA study comparing SSc eyes without any clinical signs of retinopathy and healthy control eyes in terms of foveal capillary vessel densities, a significant decrease in vessel density of inner retina has been observed in SSc eyes [145]. These reduced vessel densities were significantly correlated with subfoveal CT which was found to be thinner in the SSc group. The authors suggested OCTA as a useful tool for evaluating early vascular alterations in patients with SSc [145]. Another recent OCTA study showed a decrease in choriocapillary flow area of SSc patients. Furthermore, the authors stated that vascular density was more decreased with the presence of retinopathy. As OCTA is effective in evaluating choriocapillaris density, but not the Haller and Sattler’s layers, they assumed that the decrease in CT was mainly caused by these two layers [146].
  *Dermatomyositis and polymyositis are* idiopathic inflammatory myopathies characterized by chronic, progressive muscle inflammation and weakness. Dermatomyositis also includes a broad spectrum of cutaneous manifestations [147]. Ocular involvement, which is very rare, may potentially affect any structure of the eye. Besides eyelid and extraocular muscle involvement, several posterior segment findings including retinal hemorrhages, proliferative retinal vasculopathy, cotton-wool spots, frosted branch angiitis, central retinal artery occlusion, serous retinal detachment, optic neuropathy and choroidopathy have been reported [147–151]. Hotta reported a case with polymyositis who presented with bilateral choroidopathy and serous retinal detachment. Application of laser photocoagulation at FA leakage points resulted with resolution of serous retinal detachment in one eye. In the other eye not treated with laser, bullous retinal detachment and multifocal disciform choroidal nodules developed leading to severe visual loss [148]. As the patient was receiving corticosteroid therapy for the associated interstitial pneumonia, the author suggested corticosteroid induced choroidopathy to be considered in the differential diagnosis. However, multifocal choroidopathy observed in one eye was considered as focal inflammation of the choriocapillaris rather than being a corticosteroid related condition [148].

## Conclusions

Because of its high vascular component, the choroid may be involved in systemic inflammatory diseases affecting the vascular system. Choroidal involvement may have been underappreciated in past studies using FA. While overt signs of choroidal involvement such as serous retinal detachments, deep placoid lesions, or the Amalric sign may be observed and confirmed by multimodal imaging, subclinical involvement may also be revealed by especially ICGA and OCT imaging of the choroid. Changes in CT have been found in a multitude of studies of patients with systemic vasculitides; however, there are conflicting results and longitudinal studies are required to establish CT as a clinically useful biomarker. Advances in imaging of the choroid, especially in noninvasive imaging by OCTA that allows widefield scans of medium and large choroidal vessels will improve our understanding of choroidal vasculopathies in systemic vasculitic diseases.

## Data Availability

Not applicable.

## Abbreviations

ANCA: Antineutrophil cytoplasmic antibody
APMPPE: Acute posterior multifocal placoid pigment epitheliopathy
AVV: Antineutrophil cytoplasmic antibody associated vasculitis
BD: Behçet’s disease
CHCC: Chapel Hill Consensus Conference
CS: Cogan’s syndrome
CSCR: Central serous chorioretinopathy
CT: Choroidal thickness
CV: Cryoglobulinemic vasculitis
CVI: Choroidal vascularity index
EDI-OCT: Enhanced depth imaging optical coherence tomography
EGPA: Eosinophilic granulomatosis with polyangiitis
ET-1: Endothelin-1
FA: Fluorescein angiography
GBM: Glomerular basement membrane
GCA: Giant cell arteritis
GPA: Granulomatosis with polyangiitis
HCQ: Hydroxychloroquine
HUV: Hypocomplementemic urticarial vasculitis
ICGA: Indocyanine green angiography
IgAV: IgA vasculitis
KD: Kawasaki disease
LVV: Large vessel vasculitis
MPA: Microscopic polyangiitis
MVV: Medium vessel vasculitis
OCT: Optical coherence tomography
OCTA: Optical coherence tomography angiography
PAN: Polyarteritis nodosa
RA: Rheumatoid arthritis
RP: Raynaud’s phenomenon
RPE: Retinal pigment epithelium
SD-OCT: Spectral domain optical coherence tomography
SLE: Systemic lupus erythematosus
SOV: Single-organ vasculitis
SS-OCT: Swept source optical coherence tomography
SSc: Systemic sclerosis
SVV: Small vessel vasculitis
TA: Takayasu arteritis
VKH: Vogt-Koyanagi-Harada
VVV: Variable vessel vasculitis
